# Case Report: Metastasis to pleura and mediastinal lymph nodes from breast secretory carcinoma: 10 years follow-up

**DOI:** 10.3389/fonc.2025.1641375

**Published:** 2025-09-23

**Authors:** Fengbo Huang, Jing Zhao, Jie Lian, Bo Hong, Xiaoyan Yu, Xiaojing Ma, Jiabin Lai, Wei Qian, Jinglian Tu, Fuming Qiu, Hong Zou, Jian Huang, Jinfan Li

**Affiliations:** ^1^ Department of Pathology, Second Affiliated Hospital, Zhejiang University School of Medicine, Hangzhou, China; ^2^ Key Laboratory of Tumor Microenvironment and Immune Therapy of Zhejiang Province, Second Affiliated Hospital, Zhejiang University School of Medicine, Hangzhou, China; ^3^ Department of Medical Oncology, Second Affiliated Hospital, Zhejiang University School of Medicine, Hangzhou, China; ^4^ Cancer Institute, Key Laboratory of Cancer Prevention and Intervention, Ministry of Education, The Second Affiliated Hospital, Zhejiang University School of Medicine, Hangzhou, Zhejiang, China; ^5^ Department of Pathology, Shangyu People’s Hospital, Shaoxing, China; ^6^ Department of Radiology, Second Affiliated Hospital, Zhejiang University School of Medicine, Hangzhou, China; ^7^ Cancer Center, Zhejiang University, Hangzhou, China; ^8^ The Department of Breast Surgery, Second Affiliated Hospital, Zhejiang University School of Medicine, Hangzhou, China

**Keywords:** breast secretory carcinoma, pleura metastasis, pathological diagnosis, ETV6::NTRK3 fusion, treatment strategies

## Abstract

Breast secretory carcinoma (BSC) is a rare low-grade malignancy that frequently exhibits the ETV6::NTRK3 fusion gene and rarely presents with distant metastasis. In this report, we describe a case of 72-year-old female diagnosed with BSC, who presented with pleura and mediastinal lymph node metastases 10 years after undergoing radical mastectomy and radiotherapy. The primary tumor was characterized by a microcystic and solid architecture, eosinophilic secretions, and vacuolated cytoplasm. Immunohistochemistry (IHC) confirmed positivity for S-100 and pan-TRK. A decade later, respiratory symptoms prompted a PET/CT scan that detected metabolically active metastases in pleura and mediastinal lymph nodes. Pleura mass biopsy revealed the same morphology and IHC profile (S-100+/pan-TRK+), confirming metastatic BSC. GATA3 and TRPS1 overlap with salivary gland tumors necessitates PET/CT for distinguishing the origin. Pan-TRK IHC and NTKR3 gene break positive confirmed secretory carcinoma. Following distant metastasis, immunotherapy combined with chemotherapy was initiated. After two treatment cycles, the patient was evaluated as having stable disease after two treatment cycles, but disease progression occurred in the later four cycles. The treatment was changed to entrectinib targeted therapy, the PET/CT re-examination showed partial remission. This case highlights the rare metastatic risk of BSC and the diagnostic necessity of integrating clinical, imaging, histopathology, and NTRK3 break-apart probe detection. Targeted therapies including NTRK3 inhibitors, show promise emphasizing the importance of multidisciplinary management for this malignancy. Awareness of BSC’s metastatic potential and tailored therapeutic strategies are crucial for optimizing outcomes.

## Introduction

Breast secretory carcinoma (BSC) is an invasive neoplasm characterized by epithelial cells that contain intracytoplasmic secretory vacuoles accompanied by extracellular eosinophilic, bubbly secretions. The neoplastic cells exhibit a variable architectural pattern and frequently demonstrate the ETV6::NTRK3 fusion gene (1). This entity accounts for less than 0.05% of all invasive breast carcinomas. Typically, it is classified as low grade and follows an indolent clinical course with a favorable prognosis (2). Metastasis beyond the ipsilateral axillary lymph nodes is rare (3, 4). In this report, we present a case involving a 72-year-old female patient diagnosed with BSC who developed metastases to the pleura and mediastinal lymph nodes ten years after undergoing radical mastectomy followed by postoperative radiotherapy. Our focus in this case study encompasses imaging findings, histopathological features, immunohistochemistry, NTRK3 gene break-apart analysis, and treatment strategies employed.

## Case description

**Figure 1:**
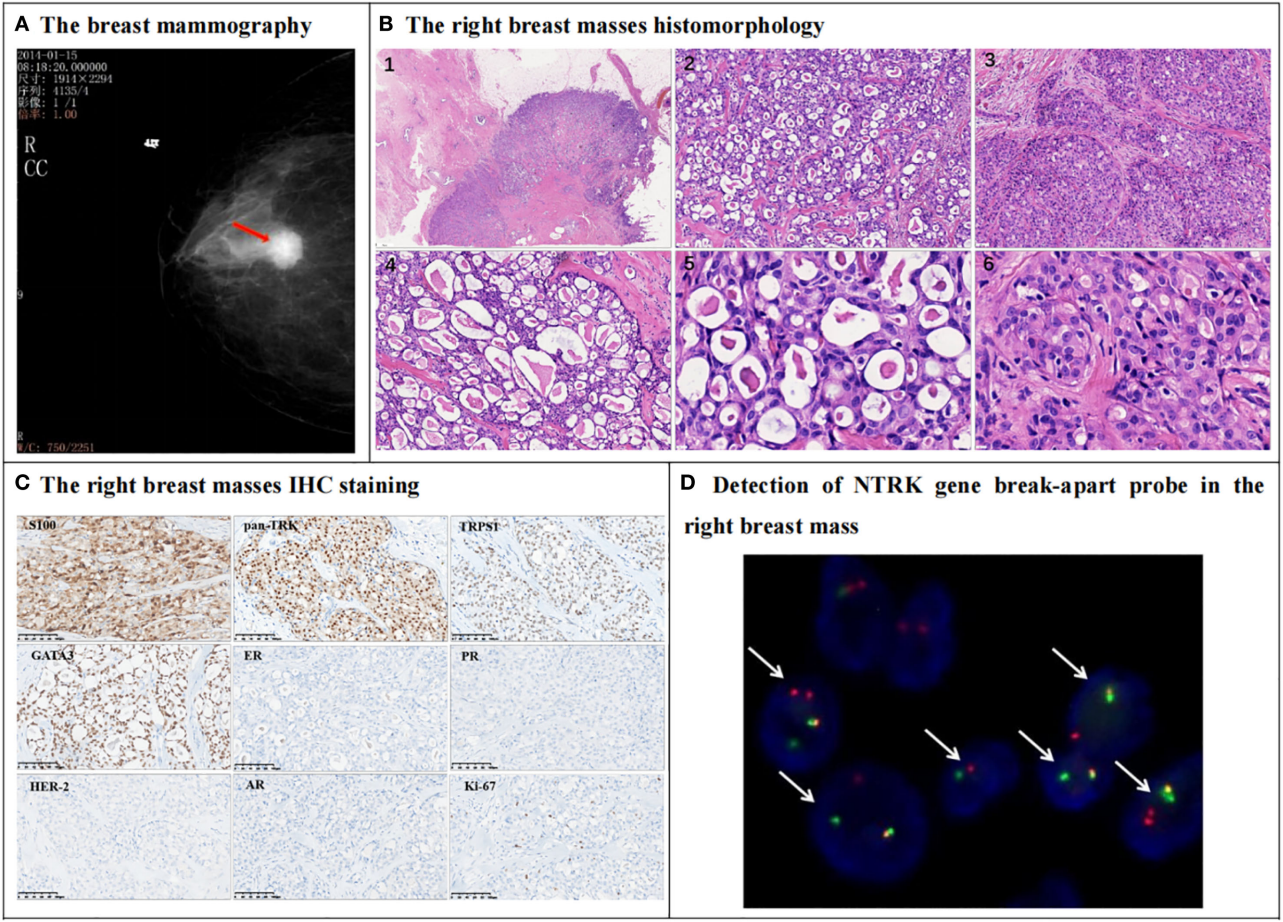
Mammography, histomorphology, immunohistochemical (IHC) and NTRK3 break-apart probe detection in the right breast mass. **(A)** The mammography indicates a high-echo nodule behind the right areola (red arrow), measuring 2.0cm by 1.5cm, and Breast Imaging-Reporting and Data System (BI-RADS) classification was 4B. **(B)** The right breast mass morphological of HE staining shows that the tumor is well-circumscribed with dilated mammary ducts observed in the adjacent tissue (B1). The tumor cells are arranged in a microcystic structure, and eosinophilic secretions can be seen inside the cysts, and collagen bundles are observed in the stroma (B2, 200X). Some tumor cells are arranged in a solid structure (B3, 200X). The cavities vary in size and their secretions are similar to thyroid colloid (B4, 200X). The nucleus is round or oval in shape, with fine chromatin and small nucleoli visible (B5, B6, 400X). **(C)** IHC shows that the tumor cells were positive for S-100, pan-TRK, TRPS1, GATA3. Estrogen receptor (ER) and progesterone receptor (PR) are negative, while HER-2 score is 0. Tumor cell was negative for androgen receptor (AR). The Ki-67 index is approximately 5%. **(D)** The FISH detection results of NTRK break-apart probe this probe show that 60% of the cells have dual-color separated fluorescence signals (white arrows), indicating a positive NTRK3 gene breakage and rearrangement (1000X).

A 72-year-old female came to the hospital ten years ago due to a mass in the right breast that had been present for one year. Mammography results indicated a high-echo nodule located behind the right areola, measuring 2.0 cm by 1.5 cm, with a Breast Imaging-Reporting and Data System (BI-RADS) classification of 4B (**Figure 1A**). Subsequently, the patient underwent a modified radical mastectomy on the right breast. Gross examination revealed a gray-white nodule measuring 2.0 cm by 1.5 cm below the areola, with a gray-white, tough-cut surface and a clear boundary.

In histopathology, the tumor was well circumscribed, and dilated mammary ducts were noted in the adjacent tissue (**Figure 1B1**). Tumor cells were arranged in a microcystic structure, and eosinophilic secretions were identified within the cysts. Collagen bundles were observed within the stroma (**Figure 1B2**). In some areas, tumor cells formed solid nests; secretory vacuoles were evident in the cytoplasm of these cells (**Figure 1B3**). The cystic spaces varied in size and contained secretions that resembled thyroid colloid (**Figure 1B4**). The tumor cells displayed bland cytologic features, with round-to-oval nuclei containing finely dispersed chromatin and small but conspicuous nucleoli. (**Figures 1B5, B6**). Mitotic figures and necrosis were not identified. Vascular invasion and lymph node metastasis were absent.

Regarding IHC staining, the tumor cells tested positive for S-100, pan-TRK, TRPS1, and GATA3. The estrogen receptor (ER) and progesterone receptor (PR) were negative, while HER-2 scored 0, consistent with a diagnosis of triple-negative breast carcinoma. The androgen receptor (AR) was also negative. The Ki-67 index was approximately 5% (**Figure 1C**). Fluorescence *In Situ* Hybridization (FISH) using the neurotrophic tropomyosin receptor kinase 3 (NTRK3) break-apart probe revealed that 60% of the breast tumor cells exhibited dual-color separated fluorescence signals (**Figure 1D**), indicative of NTRK3 breakage and potential rearrangement with a partner gene. Consequently, combining histopathology, IHC, and molecular pathology results, the patient was diagnosed with BSC, Nottingham grade 2, pT1cN0M0. Post-mastectomy, the patient did not undergo chemotherapy but was treated with radiation therapy.

**Figure 2:**
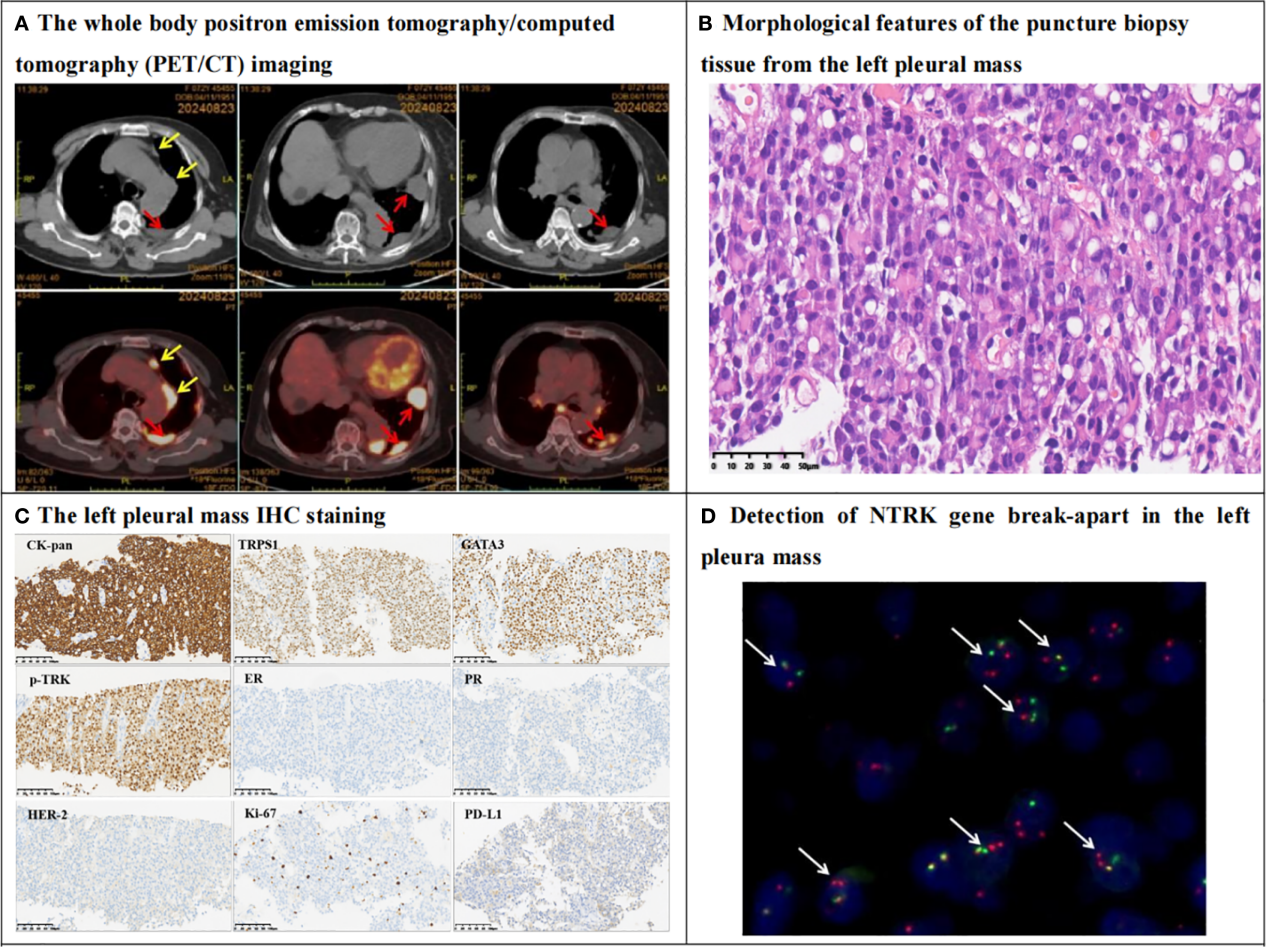
PET/CT, histomorphology, IHC and NTRK3 break-apart probe detection in the left pleura mass. **(A)** The whole body positron emission tomography/computed tomography (PET/CT) imaging revealed multiple nodules in the left pleura (red arrows), and the larger one was measured 3.3cm by1.8cm in size with standardized uptake value maximum (SUVmax) was equal to 10.19. The radioactive uptake of multiple hilar and mediastinal lymph nodes (yellow arrows) were abnormally increased, with SUVmax was equal to 10.62. **(B)** Hematoxylin-eosin staining (HE) showed that tumor cells are diffusely proliferating, with abundant eosinophilic cytoplasm or vacuolated, the tumor cells are relatively mild, with fine nuclear staining, no nucleoli, and no mitotic figures. Eosinophilic secretions are seen in the stroma (400X). **(C)** IHC shows that tumor cells express epithelial marker CK-pan, breast origin marker TRPS1, GATA3, and secretory carcinoma-specific marker pan-TRK. Tumor cells are negative for ER and PR, Her2 score is 1+, Ki67 proliferation index is approximately 10%. PDL1 CPS is 5. Envision method, 200X. **(D)** The Fluorescence *In Situ* Hybridization (FISH) of NTRK3 probe show that 75% of the tumor cells have dual-color separated fluorescence signals (white arrows), indicating a positive NTRK3 gene breakage and rearrangement (1000X).

The patient presented at the hospital with a five-day history of cough and expectoration. Chest computerized tomography (CT) revealed multiple nodules in the left pleura, the largest measuring 31.0mm by 18.0mm on Aug 16, 2024. Whole-body positron emission tomography/computed tomography (PET/CT) imaging on Aug 24, 2024 showed multiple nodules in the left subpleural region, with the largest measuring 3.3cm by 1.8cm. There was an abnormally increased radioactive uptake, with a standardized uptake value maximum (SUVmax) of 10.19. Additionally, multiple mediastinal lymph nodes exhibited an abnormally increased radioactive uptake, with an SUVmax of 10.62 (**Figure 2A**). Metastasis was considered possible. However, there was no significant abnormal increase in chromogenic uptake in the salivary glands or other body parts.

The patient underwent a biopsy of the left pleura mass. In histopathology, the tumor cells were arranged in sheets with eosinophilic cytoplasm and vacuolar changes. At high magnification, the tumor cells appeared relatively bland, exhibiting delicate nuclear staining, lacking nucleoli or mitotic figures, and eosinophilic secretions were noted in the stroma (**Figure 2B**). IHC results indicated that the tumor cells expressed the epithelial marker CK-pan, the breast-derived marker TRPS1, GATA3, and the secretory cancer-specific marker pan-TRK. Approximately 20% of the tumors were positive for S100. The tumor cells were negative for estrogen receptor (ER) and progesterone receptor (PR), the Her-2 score of IHC was 1+, and the Ki-67 proliferation index was approximately 10%. The PD-L1 combined positive score was 5 (**Figure 2C**). However, the tumor cells were negative for thyroid transcription factor-1 (TTF-1), P40, PAX-8, HepPar-1, and androgen receptor (AR). The results of the NTRK3 break-apart probe test were positive, indicative of NTRK3 breakage and potential rearrangement with a partner gene (**Figure 2D**). Based on the medical history, pleura mass biopsy, morphology, IHC, and molecular pathology, the patient was diagnosed with left pleura metastatic BSC, triple-negative breast cancer, TxNxM1.

**Figure 3:**
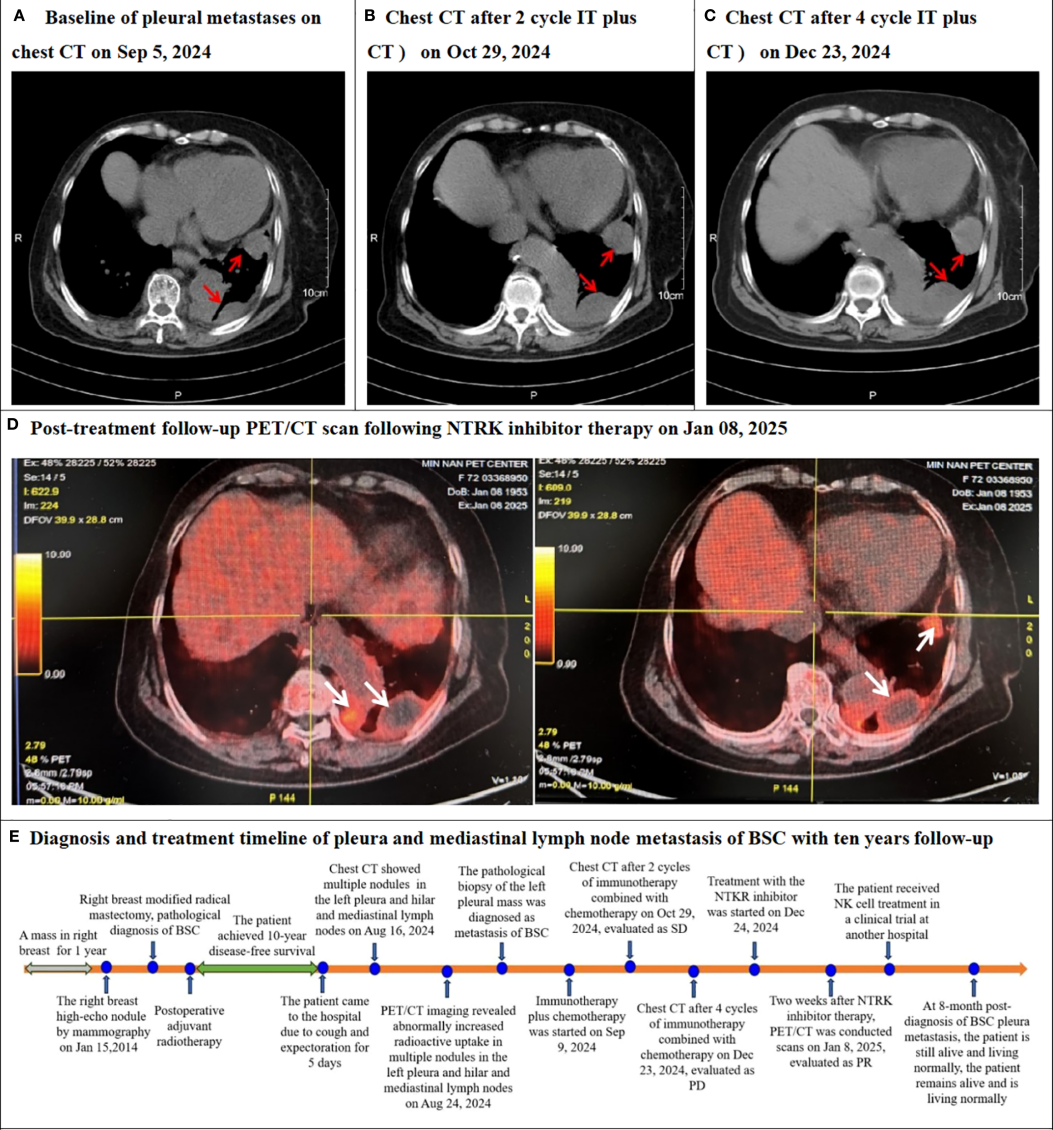
Chest CT or PET/CT after re-examination following systemic therapy and timeline of this case. **(A)** The Baseline chest CT scan performed on Sep 5, 2024, revealed left pleura masses (red arrow). **(B)** The follow-up chest CT after systemic treatment (2 cycles of immunotherapy combined with chemotherapy) on Oct 29, 2024, evaluated as stable disease (SD). **(C)** The follow-up chest CT after systemic treatment (4 cycles of immunotherapy combined with chemotherapy) on Dec 23, 2024, evaluated as progressive disease (PD). **(D)** Post-treatment follow-up the whole body positron emission tomography/computed tomography (PET/CT) scans on Jan 8, 2025, following NTRK inhibitor therapy, demonstrated reduced tumor mass and significantly diminished hypermetabolic activity (white arrows), consistent with partial response (PR). **(E)** Diagnosis and treatment timeline of pleura and mediastinal lymph node metastasis of BSC with ten years follow-up.

Upon receiving a definitive diagnosis of pleura and mediastinal lymph node metastasis of BSC, the patient began receiving immunotherapy combined with chemotherapy on Sep 9, 2024. The treatment regimen consisted of 240mg of toripalimab on day 1, combined with 100mg of albumin-bound paclitaxel on days 1 and 8. This regimen represents the standard first-line treatment for metastatic triple-negative breast cancer in China (5). Compared with a baseline of pleural metastases on chest CT on September 5, 2024, with a maximum diameter of 33.3 mm (**Figure 3A**) before immunotherapy plus chemotherapy, the patient was evaluated as having stable disease (SD), with a maximum diameter of 39.1 mm based on a chest CT scan conducted on October 29, 2024 after two cycles of therapy (**Figure 3B**). However, following an additional four cycles, the patient’s condition progressed to progressive disease (PD), with a maximum diameter of 43.9 mm by a chest CT scan on December 23, 2024 (**Figure 3C**). Due to the NTRK gene fusion, the patient was administered oral NTRK inhibitor entrectinib capsules. Two weeks into the NTRK inhibitor treatment, the patient underwent a PET/CT reevaluation on January 8 2025 (**Figure 3D**), which revealed a reduced range of glucose metabolism and a decreased SUVmax, indicating a partial response (PR). The patient ceased taking the NTRK inhibitor due to severe joint pain. Subsequently, the patient was enrolled in a clinical trial for NK cell therapy at another hospital. As of the manuscript submission date, the patient remains alive and lives normally. Diagnosis and treatment timeline of pleura and mediastinal lymph node metastasis of BSC with ten years follow-up in **Figure 3E**.

## Discussion

Only two dozen cases of distant metastasis from BSC have been reported in the English literature, and the median interval from initial diagnosis to metastatic progression was 25 months (mean: 55 months). Metastatic lesions were predominantly identified in the lungs, liver, and skeletal system, with time-to-metastasis ranging from 2.5 months to 240 months across cases (3, 4). The only case of pleura metastasis from breast secretory carcinoma in the English literature was reported by Tokunaga et al. in 1985, which was initially diagnosed as juvenile secretory carcinoma in a 13-year-old patient who involved the pleura 6 years later (6). Our described case involves a 72-year-old female patient who was initially diagnosed with BSC and subsequently developed multiple metastatic lesions in the left pleura and mediastinal lymph nodes a decade later.

In histopatholoy, the tumors display microcystic, solid, tubular, or papillary architectures. Microcystic foci closely mimic thyroid follicles and may merge into solid sheets. Most lesions show a composite pattern set within a delicate, variably fibrotic stroma and are frequently accompanied by carcinoma *in situ*. The tumor cells are polygonal and cytologically bland. They contain abundant eosinophilic to bichromophilic secretions both intracytoplasmically and within luminal spaces. The cytoplasm is granular to foamy and stains strongly with PAS, mucin, and Alcian blue. Nuclei are round to oval, exhibit mild–moderate pleomorphism, and possess inconspicuous nucleoli, and mitoses are exceedingly rare (7–10). Morphologically, secretory carcinoma must be distinguished from a group of low-grade neoplasms that share microcystic, tubular or papillary patterns and abundant luminal material in the breast. these include lactational change, cystic hypersecretory hyperplasia and carcinoma, and apocrine DCIS, whereas in salivary glands the main mimics are acinic cell carcinoma, low-grade cribriform cystadenocarcinoma, low-grade mucoepidermoid carcinoma, and pleomorphic adenoma with prominent microcystic change. In our study, the tumor cells were arranged in microcystic and solid patterns, showing eosinophilic cytoplasm and extracellular eosinophilic secretions. The nuclei were mildly atypical with evenly distributed, finely granular chromatin and small nucleoli. No mitotic figures or necrosis were identified. The histological assessment was consistent with a Nottingham grade 2.

BSC are usually triple-negative, but low levels of hormone-receptor expression are not uncommon (2, 11). TRPS1 and GATA3 are negative in acinic cell carcinomas, most cribriform adenoid cystic carcinomas and neuroendocrine carcinomas, but positive in secretory carcinomas. However, TRPS1 and GATA3 can be positively expressed in secretory carcinomas of the salivary gland and the breast. Therefore, it is not possible to distinguish secretory carcinomas from the breast and salivary glands by using the two breast-specific markers (12). If diffuse and/or at least focal intense nuclear staining was considered as the threshold for a positive result, pan-TRK had an immunosensitivity of 83.3% and a specificity of 100% (13). The findings of Ye Q et. Al (14). support the use of pan-TRK IHC to distinguish BSC from triple-negative histologic types such as adenoid cystic carcinoma, apocrine carcinoma, and acinic cell carcinoma. In our case, the primary breast lesions and the left pleura metastases were triple-negative, positive for TRPS1, GATA3, pan-TRK, and NTRK3 break-apart test, which was consistent with the diagnosis of secretory carcinoma. Based on the patient’s history of BSC, multiple pleura and mediastinal lymph nodes tumors, and no tumors in other parts of the body on PET/CT, the patient was finally diagnosed with the pleura and mediastinal lymph nodes metastasis of BSC.

Gong P et. al (2). analyzed 190 BSC patients in Surveillance, Epidemiology and End Results (SEER) program and showed that the breast cancer-specific survival rate (BCSS) was significantly better in the breast-conserving surgery plus radiotherapy group than in the mastectomy group. Qi M et. al (15). reported that adjuvant chemotherapy is recommended for those with a high lymph-node ratio BSC. In our study, the patient underwent a single mastectomy and axillary lymph node dissection for breast cancer 10 years ago with postoperative radiotherapy. When the disease progressed to the left pleura and mediastinal lymph nodes, the patient underwent immunotherapy combined with chemotherapy according to the treatment protocol for advanced triple-negative breast cancer in China (16). After two treatment cycles, the patient was evaluated as having stable disease after two treatment cycles, but disease progression occurred in the later four cycles. Due to the presence of NTRK3 fusion positivity (17), the treatment was changed to entrectinib targeted therapy, and the PET/CT re-examination showed partial remission. At 9 month post-diagnosis of BSC pleura metastasis, the patient is still alive and living normally.

In conclusion, pleura and mediastinal lymph nodes metastasis of BSC is quite infrequent. Clinicians should therefore keep this possibility in mind whenever patients present with compatible symptoms and multiple pleural nodules. The clinical history of BSC, PET/CT examination, histomorphology (eosinophilic or vacuolar cytoplasm, eosinophilic secretion), pan-TRK, S100, GATA3, TRPS1 IHC staining, and NTRK3 break-apart detection are instrumental in diagnosing BSC. Should the disease progress, oral NTRK inhibitor therapy may benefit to patients.

## Data Availability

The original contributions presented in the study are included in the article/supplementary material. Further inquiries can be directed to the corresponding authors.
